# Diagnosis of Carrion’s Disease by Direct Blood PCR in Thin Blood Smear Negative Samples

**DOI:** 10.1371/journal.pone.0092283

**Published:** 2014-03-20

**Authors:** Juana del Valle Mendoza, Wilmer Silva Caso, Carmen Tinco Valdez, Maria J. Pons, Luis J. del Valle, Verónica Casabona Oré, Denisse Champin Michelena, Jorge Bazán Mayra, Víctor Zavaleta Gavidea, Martha Vargas, Joaquim Ruiz

**Affiliations:** 1 Facultad de Ciencias de la Salud. Universidad Peruana de Ciencias Aplicadas - UPC, Lima, Peru; 2 Instituto de Investigación Nutricional, Lima, Peru; 3 Barcelona Centre for International Health Research (CRESIB, Hospital Clínic - Universitat de Barcelona), Barcelona, Spain; 4 Universidad Politécnica de Catalunya (UPC), Barcelona, Spain; 5 Dirección Regional de Salud de Cajamarca (DIRESA-Cajamarca), Cajamarca, Peru; 6 Fundación Clinic, IDIBAPS, Hospital Clinic i Provincial de Barcelona, Barcelona, Spain; The University of Hong Kong, Hong Kong

## Abstract

*Bartonella bacilliformis* is the etiologic agent of Carrion's disease. This disease has two well established phases, the most relevant being the so called Oroya Fever, in which *B. bacilliformis* infect the erythrocytes resulting in severe anemia and transient immunosuppression, with a high lethality in the absence of adequate antibiotic treatment. The presence of *B. bacilliformis* was studied in 113 blood samples suspected of Carrion’s disease based on clinical criteria, despite the absence of a positive thin blood smear, by two different PCR techniques (using Bartonella-specific and universal *16S rRNA* gene primers), and by bacterial culture. The specific *16S rRNA* gene primers revealed the presence of 21 *B. bacilliformis* and 1 *Bartonella elizabethae*, while universal primers showed both the presence of 3 coinfections in which a concomitant pathogen was detected plus *Bartonella*, in addition to the presence of infections by other microorganisms such as *Agrobacterium* or *Bacillus firmus*. These data support the need to implement molecular tools to diagnose Carrion’s disease.

## Introduction

The genus *Bartonella* includes at least 24 different species of bacteria. Currently, at least 9 of these microorganisms have been associated with infectious diseases in humans and some, such as *Bartonella bacilliformis*, *Bartonella quintana* or *Bartonella henselae* have been fully identified as causative agents of disease [1], [2].

*B. bacilliformis* are Gram-negative cocco bacilli which infects red blood cells and endothelial cells, being the etiologic agent of Bartonellosis also known as Carrion's disease, which is a biphasic disorder. Thus, two well established phases have been described in this disease, the first being the acute phase, the so-called Oroya Fever, in which *B. bacilliformis* microorganisms infect the erythrocytes resulting in severe anemia and transient immunosuppression [3], [4] which may result in death in the absence or delay of adequate treatment. The second or chronic phase, also named ‘Verruga Peruana’ or ‘Peruvian warts’, is characterized by the development of nodular dermal eruptions [5]. Asymptomatic carriers have also been described in endemic areas.

Although *B. bacilliformis* can be cultured from blood samples, including those stored at 4°C over a long period of time [6] or biopsies of eruptive or subcutaneous nodules [7], the growth of these bacteria has been shown to be difficult and slow, requiring at least 2–6 weeks of culture.

**Figure 1:**
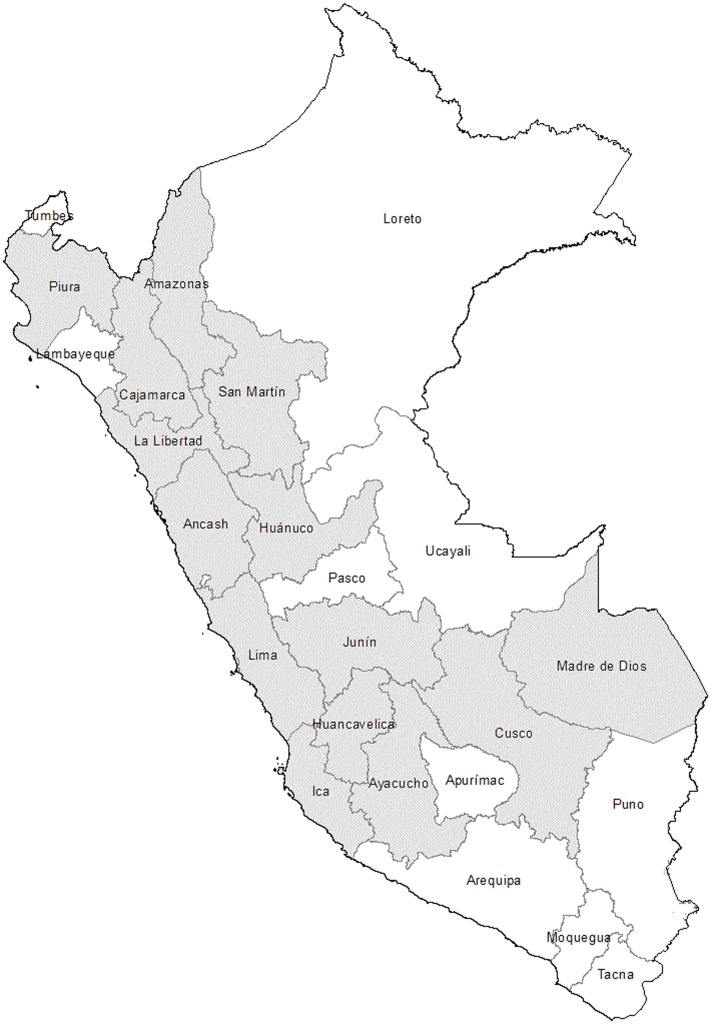
Geographical distribution of Carrion’s disease in Peru. *Carrion’s disease has also been sporadically described in other departments such as some specific areas of Loreto.

This pathogen is transmitted by the bite of members of the genus *Lutzomyia* including *L. verrucarum*, *L. peruensis* and *L. pescei* [8], which are distributed on the Western side of the Cordillera of the Andes, affecting Ecuador, Colombia and Peru, and is also sporadically reported in Bolivia and Chile [8] at 800 to 3200 meters above sea level [3], [9], [10]. Carrion’s disease is endemic and widespread in Peru, being present in at least 14 of the 24 departments of the country: Piura, Cajamarca, Amazonas, San Martin, La Libertad, Ancash, Lima, Huancavelica, Huánuco, Ica, Junín, Ayacucho, Madre de Dios and Cuzco; with the department of Ancash, being the most important endemic area (Figure 1) [1], [7], [11]. At present, human Bartonellosis is considered as an emerging/reemerging disease which is influenced by climate changes caused by the “Niño phenomenon” [7], [12].

In endemic areas the diagnosis of the acute phase of the disease is currently mainly based on clinical symptoms and by the detection of the bacteria in peripheral blood smears, which is a useful, extensively used technique in health centers, especially in remote areas. Nonetheless, this method is “expertise-dependent” having a low sensitivity ranging from 24–36% [8]. Despite having a high specificity, the microbiological culture has a very low sensitivity because of the aforementioned difficulty to culture this microorganism. Moreover, the clinical utility of this method is limited because of the slow growth of these bacteria [7]. In this context, the development of various new molecular biology techniques for rapid diagnosis of this pathogen is of crucial relevance [8].

The aim of this study was therefore to develop and implement a PCR method as a rapid diagnostic tool for the detection of *Bartonella* spp. in blood samples, by analyzing a series of clinically suspected *B. bacilliformis* infections with a negative thin blood smear.

## Methods

### Patients and sampling

One hundred thirty-six venous blood samples of patients submitted to the Regional Laboratory of Cajamarca – Peru from January 2010 to December 2011 with clinical suspicion of Carrion’s disease but with a negative *B. bacilliformis* thin blood smear, were sent to the Instituto de Investigaciones Nutricionales (IIN, Lima, Peru) for diagnosis by molecular tools. The results obtained were reported to physicians for clinical management of the patients.

Relevant medical information of the patients was recorded on a form developed by the Instituto Nacional de Salud (INS) - Peru. Venous blood was collected in tubes containing EDTA and citrate and sent to the IIN facilities where they were stored at 4°C until processing.

Samples were collected for diagnosis purposes following an explanation to each of the participants by the physician responsible for the health center, in all cases, an informed consent for testing was obtained from patients, samples were coded to preserve the anonymate of the patients, and results were timely reported to local physicians.

Molecular diagnostic analysis of these samples was carried out as part of febrile syndromic surveillance of the Cajamarca department, conducted by the Regional Directorate of Health (DIRESA-Cajamarca) which is the regulatory center under a collaborative agreement with the IIN to improve the diagnostic of the Carrion Disease.

The Cajamarca Regional Directorate has no ethics committee. thus, despite the encoding and the diagnostic nature of the samples, and them exempt to be approved by an Human Subjects Committe, the studies were submitted, revised and approved by the Ethics and Research Committees of the Hospital Clinic of Barcelona.

### Bacterial culture conditions

Microorganisms were cultured as previously described [6], [13] with slight modifications. Briefly, 2 ml of the blood sample were grown in Columbia agar medium supplemented with 10% sheep blood and incubated at 28°C for approximately 45 days under anaerobic conditions. The plates were visually inspected at 24, 48 and 72 hours to detect contamination, and then each 7 days for bacterial growth. Colonies were identified based on the characteristic morphology and Gram staining. *Bartonella* spp. were confirmed by amplification and sequencing of a 438 bp region of the *16S rRNA* gene using *16S rRNA* gene-specific primers for the genus *Bartonella.* When no amplification product was obtained, *16S rRNA* gene-universal primers were used, and the product obtained was also sequenced. The conditions of both cases are described below.

All the bacteria isolated and included in the study are disposable for scientific non-commercial purposes.

### DNA extraction

The DNA was extracted from 200 μl of blood samples using a commercial extraction kit (High Pure Kit Preparation template, Roche Applied Science, Mannheim, Germany). Bacterial DNA obtained after extraction was eluted in 100 μl of nuclease free water and then processed or stored at –20°C until use.

### Amplification of a *Bartonella* spp.-specific *16S rRNA* gene fragment

A 438 bp fragment of the *16S rRNA* gene was amplified both in blood samples and in the microorganisms isolated adapting *Bartonella* spp. *16S rRNA* gene-specific primers (Table 1) and PCR conditions designed by Garcia-Esteban *et al.* [14]. The final volume of the PCR mixture was 50 μl, distributed as follows: 25 μl of enzyme mix (Taq polymerase, 2.5 mM MgCl_2_, 15 mM Tris/HCl pH 8.3, 50 mM KCl, 200 μM of each deoxynucleotide), 20 pmol of each primer (Macrogen, Seoul, Korea), and 5 μl of DNA extraction. Thus, the PCR conditions were: 95°C for 10 min, followed by 45 cycles of 94°C for 1 min, 55°C for 1 min and 72°C for 1 min, with a final elongation of 10 min at 72°C. The amplified DNA products were analyzed by gel electrophoresis on 2% agarose (FMC, Rockland, ME) gel containing ethidium bromide (3 mg/L). Amplified products were gel recovered, purified (SpinPrep™ Gel DNA Kit, San Diego, USA) and sent to be sequenced (Macrogen, Seoul, Korea).

**Table 1:** The primers and conditions used in this study.

Primers	Sequence	Size (bp)	Reference
P24Emod	CCTTCAGTTMGGCTGGATC	438	[14]
16S-R	GCCYCCTTGCGGTTAGCACA†		
			
8F	AGAGTTTGATCCTGGCTCAG	1503	[15]
1510R	GGTTACCTTTGTTACGACTT		

† The primer described in reference 14 has three more bases (GCCYCCTTGCGGTTAGCACAGCA).

### Amplification of *16S rRNA* gene fragments using universal primers

This protocol was performed using the aforementioned PCR conditions and the universal primers described previously [15] and was applied in all blood samples as well as in microorganisms in which no positive PCR was obtained using *Bartonella* spp.-specific primers.

### Data analysis

DNA sequences were analyzed with the BLAST analysis tool and compared with the GenBank database. Statistical significance was established using the Fisher exact test. Differences were considered as significant with a p-value <0.05.

## Results

One hundred thirty-six blood samples from patients clinically diagnosed with Oroya Fever were collected, but those with no positive thin blood smear were included in the study. Of these, 23 were discarded for different reasons, including no collection of clinical data or problems with sample transportation, among others. Of the 113 samples included in the study nearly half (47%) belonged to people under 20 years of age, while no significant differences in sex were observed. PCR confirmed that *B. bacilliformis* infections were mainly detected in men (13 cases, 62%), while only 7 women (33%) were positive. Additionally, in one case data regarding sex was not specified. In positive cases the age ranged from 3 to 55 years, being mainly recovered from the age range from 26–35 years (35%), followed by age groups of 19–25 and <10 years (23% and 22%, respectively) (Table 2).

**Table 2:** Characteristics of the population studied.

*Age (years old)*	Frequency	Percentage (%)	PCR-positive†
≤ 10	18	15.93	4 (22.0%)††
11 – 18	29	25.66	4 (13.8%)
19 – 25	13	11.50	3 (23.0%)
26 – 35	20	17.70	7 (35.0%)
36 –45	17	15.04	1 (5.8%)
46 – 55	7	06.20	1 (14.3%)
≥ 56	8	07.08	1 (12.5%)
No data	1	00.88	1 (100%)
***Area of Origin***			
Cajamarca	109	96.46	
Lima	2	01.77	
Ancash	2	01.77	
***TOTAL***	**113**	**100%**	

† Positive samples using specific primers for *Bartonella 16S rRNA*.

†† Includes a *B. elizabethae* isolate.

The main clinical presentations reported by clinicians were general discomfort (57.66%), fever (52.25%) and headache (51.30%), in addition to other symptoms including myalgia and joint pain, among others (Table 3). Regarding the PCR-confirmed cases the main symptoms reported were headache (76.19%) followed by fever, chills and joint pain (66.70%). The use of the Fisher test showed the presence of significant differences among clinical symptoms between PCR confirmed and non confirmed *Bartonella* cases. Thus, loss of appetite, pollakiuria (p<0.0001), chills, joint pain, pallor, cough and ictericia (p<0.05) were significantly more frequent among PCR-confirmed *Bartonella* cases. Additionally, a trend towards significance (p = 0.066) was observed with respect to abdominal pain in patients with confirmed *B. bacilliformis*. No data regarding erythrocyte counts were available.

**Table 3:** Signs and symptoms.*

	Total Patients (N: 113)		PCR-positive *B. bacilliformis cases* (N: 21)		PCR-negative cases (N: 92)	
Signs and symptoms	No	Percentage (%)	No	Percentage (%)	No	Percentage (%)
Discomfort	64	57.66	12	57.14	52	56.52
Fever	58	52.25	14	66.70	44	47.83
Headache	57	51.30	16	76.19	41	44.57
Chills	55	49.54	14^††^	66.70	41^††^	44.57
Myalgia	50	44.70	12	57.14	38	41.30
Joint pain	48	43.24	14^††^	66.70	34^††^	36.96
Pallor	36	32.43	12	57.14	24	26.08
Abdominal pain	34	30.63	10	47.62	24	26.08
Nausea	30	27.02	6	28.57	24	26.08
Dizziness	24	21.61	2	09.52	12	13.04
Cough	24	21.61	9^††^	42.86	15^††^	16.30
Vomiting	21	18.90	3	14.29	18	19.56
Diarrhea	21	18.90	6	28.57	15	16.30
Loss of appetite	13	11.70	13†	61.90	0†	0
Sore throat	13	11.70	0	00.00	13	14.13
Dyspnea	13	11.70	3	14.29	10	10.87
Pollakiuria	10	09.00	10†	47.62	0†	0
Ictericia	10	09.00	5^††^	23.81	5^††^	5.43
Warts	7	06.30	1	04.76	6	6.52
Dysuria	7	06.30	2	09.52	5	5.43
Others¥		Less than1%				

† p<0.001††p<0.05 (Statistical significance established using the Fisher exact test).

* Symptoms considered on the basis of standard solicited data for the surveillance of Carrion’s disease.

¥Others: cyanosis, drowsiness, itching, back pain, chest pain, petechiae, congestion.

The samples were analyzed using two different techniques: bacterial culture and two PCR schemes. Thirteen samples (11.71%) were culture positive. Of these, 11 were identified as *B. bacilliformis*, one as *Bartonella elizabethae* and the remaining sample was identified to be *Bacillus firmus.* Twenty-two (19.46%) blood samples were positive on amplification of a specific fragment of the *Bartonella 16S rRNA* gene; of these 21 were *B. bacilliformis* (18.58%) and 1 *B. elizabethae* as confirmed when sought in GenBank as showing 100% of identity with sequences NR_074203.1 (*B. bacilliformis*) and L01260.1 (*B. elizabethae*). Regarding bacterial culture, amplification of a specific fragment of the *Bartonella 16S rRNA* gene showed a sensitivity of 100%, and a specificity of 100%. Finally, the use of universal *16S rRNA* gene primers revealed the presence of 33 (29.20%) samples presenting bacterial microorganisms: *B. bacilliformis* (18 cases; 15.93%), *Rhodoccus* spp. (3 cases; 2.70%), *B. firmus* (3 cases; 2.70%) and others such as *B.elizabethae*, *Achromobacter* spp, *Agrobacterium* spp., *Artrobacter* spp., *Bacilus cereus* or *Blastococcus* spp., among others, accounting for one each (0.9%), respectively (Tables 4 and 5).

**Table 4:** Comparison of the techniques used to identify *Bartonella* spp.

	Microbiological culture N(%)	Direct blood PCR of *16S rRNA* gene Bartonella spp. N(%)	Direct blood PCR of *16S rRNA* gene Universal
Positive	13 (11.5)	22 (19.5) *	33 (29.2) †
Negative	100 (88.5)	91 (80.5)	80 (70.8)
TOTAL	113 (100%)	113 (100%)	113 (100%)

* Twenty-one *B. bacilliformis*, 1 *B. elizabethae.*

† Also include other microorganisms: *Rhodoccus* spp. (3 isolates) *Bacillus firmus* (3 isolates), *Achromobacter xylosoxidans,* *Agrobacterium* spp., *Artrhobacter* spp, *Bacillus cereus*, *Blastococcus* spp., *Pseudomonas putida* and *Staphylococcus pettenkoferi*.

**Table 5:** Information about culture and direct blood PCR in positive Samples.

		Direct Blood PCR	
Sample	Culture*	*16S rRNA*-*Bartonella*	*16S rRNA* Universal
1	+	*B.bacilliformis*	*B. bacilliformis*
2	+	*B.bacilliformis*	*B. bacilliformis*
3	+	*B.bacilliformis*	*B. bacilliformis*
8	+	*B.bacilliformis*	*B. bacilliformis*
11	–	*B.bacilliformis*	*Arthrobacter* sp
14	+	*B.bacilliformis*	*B. bacilliformis*
22	+	*B.bacilliformis*	*B. bacilliformis*
28	+	*B. elizabethae*	*A.xylosoxidans*
32	+	*B.bacilliformis*	*B. bacilliformis*
35	+	*B.bacilliformis*	*B. bacilliformis*
36	+	*B.bacilliformis*	*B. bacilliformis*
38	–	*B.bacilliformis*	*B. bacilliformis*
39	–	*B.bacilliformis*	*B. bacilliformis*
43	–	*B.bacilliformis*	*B. bacilliformis*
44	+	*B.bacilliformis*	*B. bacilliformis*
48	–	*B.bacilliformis*	*B. bacilliformis*
50	–	–	*Achromobacter* spp.
54	+	*B.bacilliformis*	*B. firmus*
56	–	–	*Rhodococcus* spp
59	–	–	*Rhodococcus* spp
61	–	–	*Bacillus cereus*
67	–	–	*Agrobacterium* spp
68	–	–	*Bacillus firmus*
76	+	–	*Bacillus firmus*
77	–	–	*Blastococcus* spp.
78	–	–	*S. pettenkoferi*
89	–	–	*Rhodococcus* spp.
100	–	*B.bacilliformis*	*B. bacilliformis*
103	–	*B.bacilliformis*	*B. bacilliformis*
124	–	–	*P. putida*
125	–	*B. bacilliformis*	*B. bacilliformis*
129	–	*B. bacilliformis*	*B. bacilliformis*
136	–	*B. bacilliformis*	*B. bacilliformis*

* All positive cultures but No. 76 were identified as *B. bacilliformis* or *B. elizabethae* (culture No. 28). Culture 76 was identified as *Bacillus firmus,* in accordance with direct blood PCR assays.

The analysis of concordance between the techniques used showed that in all cases in which *Bartonella* positive culture was obtained, both PCR schemes also detected its presence. Besides, in 9 cases the PCR techniques detected the presence of a *Bartonella* spp. in the absence of a positive culture. In all cases but three both PCR techniques detected *B. bacilliformis* in the same samples. In these three non-concordant cases *B. bacilliformis* was detected amplifying the specific *16S rRNA* fragment, while another microorganism was identified using the *16S rRNA* universal primers being interpreted as coinfections. Thus, analysis of the results showed the presence of *B. bacilliformis* plus *B.firmus*, *B.bacilliformis* plus *Artrhobacter* spp. and *B. elizabethae* plus *Achromobacter xylososidans*.

## Discussion

The reporting of *B. bacilliformis* in Peru is based on the analysis of microbiological culture or thin blood smear. This last technique is the most frequently used in endemic areas, but it has a series of limitations including a low sensitivity. Laboratory confirmation by culture and isolation of the causative organism is generally considered to be the gold standard for the diagnosis of most bacterial infections. However, *Bartonella* spp. are fastidious and fragile organisms which are difficult to culture and require a period of growth of 15 to 45 days, depending on the species.

Thus, the use of other techniques for adequate disease identification is necessary [8], [14]. Some techniques have been proposed, including ELISA, immunoblotting, indirect fluorescence antibody tests and PCR [8], [14]. However, some of these techniques present a series of limitations including false positive results. It has been proposed the use of Real Time PCR in the diagnosis of infections due to different *Bartonella* species [16]–[18], however, as other aforementioned tests, this technique may be expensive and cumbersome for routine implementation in the main endemic areas, most of which are located in remote low-income remote regions of Peru.

On the other hand, PCR for the diagnosis of *Bartonella* spp. infections is not-cumbersome and is a sensitive, specific and rapid molecular technique, with a relatively low cost [8. 14]. Additionally, if combined with restriction analysis, may result in the exact specie identification [19]. Nonetheless the characteristics of PCR do not allow its implementation in low-income rural areas, but it does have potential for use in regional healthcare centers which may act as reference centers providing early results to local physicians. Therefore, PCR may be an alternative to improve the diagnosis of infection in these areas [20]. Indeed, the use of a specific PCR may result in better diagnosis of *Bartonella* infections, while the use of universal *16S rRNA* gene primers may facilitate the detection of other bacterial pathogens causing febrile syndromes as well as coinfections between *Bartonella* and other microorganisms such as what occurred in the present report. Thus, the combined use of the present two PCR techniques may also have the advantage of detecting both *B. Bacilliformis* as well as other microorganisms present either as the main infective cause or as a concomitant opportunistic infection. In fact, the use of universal *16S rRNA* gene primers has been explored in order to facilitate diagnosis of central nervous system infections [21].

A series of samples clinically suspected of having *B. bacilliformis*, but with no positive blood smear, were collected in the area of Cajamarca. In these samples the amplification of a specific fragment of the *B. bacilliformis 16S rRNA* gene showed the presence of this microorganism in 21 out of 113 samples, again showing both the strong limitations of the thin blood smear to detect *B. bacilliformis* and the need to implement more efficient techniques for achieving correct diagnoses in endemic areas. This result is in accordance with previous studies which have demonstrated that the PCR technique detects *B. bacilliformis* with a higher sensitivity than blood smear [22].

It is of note that the presence of *B. elizabethae* in a case of suspected Oroya Fever, suggesting the potential role of other members of the *Bartonella* genus to cause Oroya Fever-like illness, as has been previously described in the case of *Bartonella rochalimae* [23], and supporting the descriptions of patients with compatible symptoms in other distant areas, such as Thailand or Guatemala [8].

A series of other pathogens was also detected in blood samples using universal *16S rRNA* primers, including one *Agrobacterium* spp., an environmental microorganism with a close phylogenetic relation to the *Bartonella* genus. This microorganism has been described as causing illness in immunocompromised patients [24]. Most of the remaining microorganisms detected are also considered to be opportunistic pathogens or have only been detected as causing infections in immunocompromised patients [25]–[28]. The presence of this kind of pathogen, together with the ability of *B. bacilliformis* to produce transient immunosuppression [4] which facilitates the presence of concomitant infections, may suggest the presence of undetected *B. bacilliformis*. Although no direct data on erythrocyte counts were reported, the clinical symptoms of the suspicious PCR-positive *Bartonella* cases were suggestive of a status of anemia. Patients with confirmed *Bartonella* present a significantly higher prevalence of paleness and ictericia. Regarding other symptoms a series of significant differences were also observed between PCR-positive *Bartonella* and PCR-negative *Bartonella.* These differences together with those in ictericia and pallor support the contention that these two populations represent two different infectious processes and therefore support the utility of the amplification of a specific fragment of the *Bartonella 16S rRNA* gene to achieve a more precise etiologic diagnosis. Further studies in a larger number of Carrion disease-positive cases are needed to confirm these clinical findings.

*B. bacilliformis* was detected in three samples by amplifying the specific fragment of the *Bartonella 16S rRNA* gene, while another microorganism was detected using the universal *16S rRNA* primers. Although the possibility of contamination can not be ruled out, the lack of a negative control demonstrating a contamination band leads to a possible scenario of a coinfection. This differential amplification might be related to the specific amount of each pathogen in blood as well as to the intracellular nature of *B. bacilliformis*. Both of these factors may result in better amplification of one pathogen with respect another. Although DNA sequencing is not a feasible technique to be implemented in most endemic rural areas, the present data suggest that the use of both techniques may provide a better diagnosis.

In the present report, most of the cases of suspected Oroya Fever belonged to young patients. This fact may be related to the age-structure of the populations of the areas as well as the partial immunity developed after *B. bacilliformis* exposure which may provide a degree of protection against further infections by this microorganism as has been previously proposed [1].

In summary, the present data show the urgent need to introduce better diagnostic tools in the areas in which Carrion’s disease is endemic in order to improve the diagnosis and management of this illness and to avoid possible misdiagnoses.
